# From the Psychiatrist’s Couch to Induced Pluripotent Stem Cells: Bipolar Disease in a Dish

**DOI:** 10.3390/ijms19030770

**Published:** 2018-03-08

**Authors:** Anke Hoffmann, Vincenza Sportelli, Michael Ziller, Dietmar Spengler

**Affiliations:** Max Planck Institute of Psychiatry, Translational Psychiatry, 80804 Munich, Germany; hoffmann@psych.mpg.de (A.H.); vincenza_sportelli@psych.mpg.de (V.S.); michael_ziller@psych.mpg.de (M.Z.)

**Keywords:** bipolar disease, patient-specific iPSC, early neurodevelopment, lithium, neuronal excitability, calcium signaling

## Abstract

Bipolar disease (BD) is one of the major public health burdens worldwide and more people are affected every year. Comprehensive genetic studies have associated thousands of single nucleotide polymorphisms (SNPs) with BD risk; yet, very little is known about their functional roles. Induced pluripotent stem cells (iPSCs) are powerful tools for investigating the relationship between genotype and phenotype in disease-relevant tissues and cell types. Neural cells generated from BD-specific iPSCs are thought to capture associated genetic risk factors, known and unknown, and to allow the analysis of their effects on cellular and molecular phenotypes. Interestingly, an increasing number of studies on BD-derived iPSCs report distinct alterations in neural patterning, postmitotic calcium signaling, and neuronal excitability. Importantly, these alterations are partly normalized by lithium, a first line treatment in BD. In light of these exciting findings, we discuss current challenges to the field of iPSC-based disease modelling and future steps to be taken in order to fully exploit the potential of this approach for the investigation of BD and the development of new therapies.

## 1. Introduction

Bipolar disorder (BD) is a mental condition characterized by extreme mood swings that comprise emotional highs (mania or hypomania) and lows (depression) [1]. BD represents one of the most common psychiatric diseases with an estimated prevalence of 0.6% [2] and a lifetime prevalence of 1.02% [3] according to the latest BD meta-analysis. In 2015, ≈60 million people were affected by BD—almost twice as much as the number of people affected by cancer. For comparison, ≈300 million people were affected by major depressive disorder (MDD) with more women than men, and ≈21 million were affected by schizophrenia (SCZ) [4]. Recent studies show that BD is the sixth leading course of disability worldwide [5] and associates with high rates of morbidity and mortality: 23–26% of BD patients attempt suicide and it accounts for 3.4–14% of all suicide deaths [6]. Lifetime prevalence of attempted suicide is estimated at 34% and 19% for female and male BD patients, respectively [7], while the risk of completed risk is three times higher among males than females with BD. These findings suggest that suicide and suicide attempts in BD are a frequent multicausal human behavior [8]. Unfortunately, little progress has been made so far since lithium and neuroleptics were introduced in the late 50s and 60s for the treatment of BD: response rates are 50–60% [9] and no cure is widely available yet.

In this review, a literature search of articles on BD and iPSCs was performed in recognized databases such as PubMed, Scopus, and PsycINFO using the keywords “bipolar disorder”, “patient-specific iPSC”, “schizophrenia”, “neurodevelopment”, and “lithium”. Bibliographies were further scrutinized for articles and book chapters of relevance. As a result, we will describe first the multifaceted phenotype of BD, current pharmacological treatments, and recent findings on its complex genetic architecture. This provides the opportunity to introduce the concept of patient-specific induced pluripotent stem cells (iPSCs) for the study of mental disorders. Following this, we highlight recent findings and current limitations from modeling BD in the dish. Concluding, we will discuss present challenges for this in vitro approach and further steps to be taken by the field.

## 2. From the Psychiatrist’s Couch

**Figure 1:**
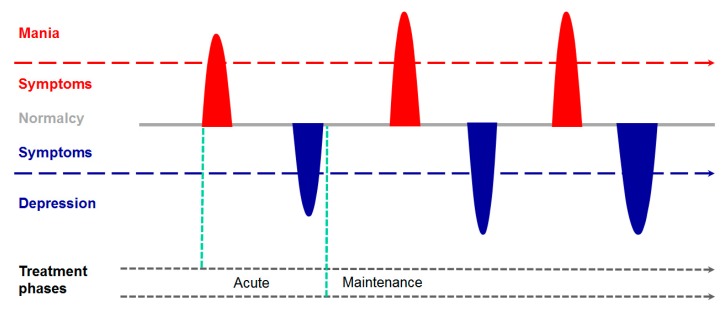
The life cycle of bipolar disease. The more severe form of bipolar disease (BD), called type I, firstly manifests a manic episode (red peak) followed by a depressive episode (blue peak). Following this first cycle, euthymic interphases (grey lines) progressively shorten until a relatively stable pattern of recurrence (single periods and/or entire cycles) develops over time. Concurrently, severity and duration of single episodes tends to increase, particularly for depressive episodes. This scheme illustrates the prototypical course of bipolar disease I whilst varying courses are common. First line treatments for manic episodes are neuroleptics and lithium. The latter can be also used for maintenance therapy of recurrent manic and/or depressive episodes.

BD typically manifests in the mid-teenage years or early 20s and persists (lifelong) with episodes of mood swings occurring rarely or multiple times a year (Figure 1). While some people may not experience emotional symptoms between episodes, most people do. Mania is characterized by a euphoric, expansive, or irritable mood that concurs with a marked increase in activity, energy or even agitation, and a decreased need for sleep. Typical symptoms comprise an exaggerated sense of well-being and self-confidence, unfamiliar talkativeness, racing thoughts, distractibility, and poor decision making; all of these symptoms cause significant impairment in interpersonal, social or work function [1]. By contrast, major depressive episodes involve a low mood of feeling empty, hopeless, worthless or guilty, marked loss of interest or feeling no pleasure in most activities, fatigue, and loss of energy. Depressed people show a decreased ability to think or concentrate leading to indecisiveness, restlessness or slowed behavior and ultimately, pondering about planning or attempting suicide. Neurovegetative symptoms include insomnia or sleeping too much and significant weight loss or gain.

The nomenclature “bipolar” evokes the impression that affected individuals stay on two separate poles of either mania or depression. At opposite, in roughly one-half of episodes manic and depressive symptoms co-occur. These mixed states are less responsive to therapy (see below) and more sensitive to manic switches and suicidal ideation [1].

BD is diagnosed as the more severe form type I, which manifest firstly a manic episode followed by a hypomanic or depressive episode, and the less severe form type II, which manifests a major depressive episode followed by at least one hypomanic, but never a manic, episode. Importantly, the early course of BD is progressive: Following a single manic episode an affected individual has an 80% risk of recurrent manic and depressive episodes accompanied by a progressive shortening of the euthymic interphase until a relatively stable pattern of recurrence evolves over time (Figure 1). In long-term studies, BD individuals were symptomatically ill in about half of the time and spent about one-third of the time with depressive symptoms, attributing thus for most of the functional impairment in BD [10,11].

Pharmacotherapy flanked by psychotherapy and psychoeducation represents the mainstay of BD treatment, both in acute episodes and maintenance therapy. Mania is the defining symptom of BD and potential antimanic medications comprise lithium, fully effective in monotherapy in 30% of the patients [12,13], conventional and atypical antipsychotics, and antiepileptics [9]. In general, all of these medications are similarly efficacious with 50% of manic individuals responding in 3–6 weeks. However, antimanic treatments additionally effective in maintenance and antidepressant therapies are preferred. Since functional recovery often trails symptom resolution by months following a major affective episode, prevention of recurrences is essential to long-term outcomes. Lithium significantly decreases the risk from both manic and depressive episodes as well from suicidality and this improvement may accumulate over time [14]. Despite ongoing development of new medications during the last two decades, none of these is curative, and trial and error remains necessary to identify the best treatment for a given individual.

Taken together, BD is a recurrent mood disorder that typically manifests in mid-adolescence to early adulthood and is a leading medical cause of disability worldwide with functional impairments persisting for months after acute episodes. Pharmacotherapy in acute episodes and maintenance therapy is effective in only half of the patients and suicide remains a major contributor to high mortality rates.

## 3. The Genetic Architecture of BD

BD has long been known to be familial with first-degree family members having on average an about ten-fold increase over the general population [15]. Since families share both genes and a common environment, this does not necessarily implicate a genetic cause. Yet, twin studies have shown that for monozygotic twins, if one is affected by BD, the other shares a 70–80% risk of manifesting BD. By contrast, this risk is only 15–20% for dizygotic twins. Heritability, as inferred from twin studies is about 85%, and thus higher than for any other psychiatric condition (e.g., SCZ 81%, Alzheimer’s disease 75%, MDD 37%, and generalized anxiety disorder 28%) [16] or any other heritable complex medical condition. 

Genetic linkage studies in BD (the largest comprising 972 unrelated families [17]), have identified several chromosomal regions (e.g., 6q, 8q, 22q, or 13q) to confer genetic vulnerability that proved to be hard to replicate yet. DNA markers do not necessarily tag the causative gene and since the identified areas contain multiple genes, linkage studies performed poorly in disclosing causative genes [15]. Likewise, genetic associations to “candidate genes” that were chosen due to their neurobiological function, localization, or both were poorly replicated with few exceptions including *BDNF* (encoding brain-derived neurotrophic factor), *5HTT* (encoding serotonin transporter), *DISC1* (encoding disrupted in SCZ), and *DAOA* (encoding D-amino oxidase activator).

Despite these limitations, recent progress in whole-exome (WE) and whole genome sequencing (WGS) has revived the interest in family studies for the identification of rarer, more penetrant, risk variants that could partly explain BD. For example, Georgi and coworkers employed several strategies to infer the role of both common and rare susceptibility alleles for BD in an exceptionally large Old Order Amish pedigree with a high incidence of the disorder [18]. This analysis uncovered an unexpected level of genetic complexity with no converging evidence for a limited number of loci conferring vulnerability to BD. Instead, BD appeared to be genetically heterogeneous due to the aggregation of different moderately frequent alleles in different sub-pedigrees. Additional studies on non-Amish families [19,20] strengthened this hypothesis: Accordingly many genes are involved in familial BD, group of risk variants differ in different families, and exonic variants of major effects are unlikely to exist. Taken together, these results suggest a polygenic mode of inheritance for familial BD.

In the past decade, technical advancements enabled the assembly of millions of single nucleotide polymorphism (SNPs) on so-called DNA chips that are now routinely used in genome-wide association (GWA) studies. Since the genome is transmitted in blocks of several thousand to several million DNA base pairs, a single SNP can tag the region around it (known as linkage disequilibrium). This allows a reduction in the number of SNPs tested for assessing efficiently common genetic variation at virtually any gene and/or regulatory region that exists in more than 1% of the population. Since many statistical tests are conducted at once, massive multiple testing correction is required, and the currently accepted threshold for a significant finding is 5 × 10^−8^. GWA studies using large case/control samples have identified several risk loci including markers near *ADCY2* (encoding adenylate cyclase 2), *ANK3* (encoding anykrin 3, a scaffolding protein), *CACNA1C* (encoding voltage-dependent calcium channel, alpha-1c subunit), *TENM4* (encoding transmembrane protein expressed predominantly in neurons), *SYNE1* (encoding spectrin repeat-containing nuclear envelope protein 1, a protein linking the plasma membrane to the actin cytoskeleton), and *TANC1* (encoding tetratricopeptide repeat and ankyrin repeat containing 1 protein, which interacts with various proteins from the synaptosom) [21,22,23,24,25]. In this context it is important to realize that contrary to Mendelian diseases, which are caused by mutations in the coding region of a gene, risk-associated SNPs frequently map to non-coding genomic regions equally represented by intergenic and intronic regions. Tag SNPs capture all the other SNPs present at the risk-associated haplotype block, but are, themselves, not necessarily the causal genetic variants that underlie the association nor do they prove a role of the gene to which they map.

At present, GWA studies and meta-analyses of GWA studies raw data from several tens of thousands individuals support the association of >40 genes with susceptibility to BD [26]. Even though, the effect size of common susceptibility SNPs is rather small (e.g., odds ratio ~ 1.2) and magnitudes recapitulate those from SCZ [27] and MDD [28,29] suggesting that additional loci remain to be identified. Consistent with the view, polygenic risk scores (i.e., the sum of associated risk alleles weighted by their estimated effect sizes) clearly support the contribution of hundreds to thousands of genetic variants, most of which are below the genome-wide significance level [25,27,30]. Additional support is provided by the high genetic correlation of BD with SCZ [31,32], where a similar genetic architecture has been detected [27,33]. Despite this progress on the genetics of BD, common risk alleles capture so far only 25–30% of the heritability in BD.

Taken together, these studies suggest a highly polygenic disease architecture with a large number of common and rare variants. Each of these variants encodes small effects that contribute incrementally to the development of BD. How these risk variants mediate molecular and cellular mechanisms that trigger disease onset and progression remains poorly understood. Hence, there is an urgent need to analyze disease genotypes in disease-relevant cellular contexts to gain insight in their molecular mode of action.

## 4. Neurodevelopmental Anomalies in BD

In 1957, Barbara Fish (for a historical review [34]), firstly suggested that faulty timing and integration of development may contribute to childhood SCZ. Some 30 years later, Weinberger [35] and Murray and Lewis [36,37] expanded on this notion and explicitly formulated the neurodevelopmental hypothesis of SCZ: Alterations in early developmental trajectories are particular sensitive to the combined effects of genes and environmental brain lesions acquired during pregnancy or postnatally and ultimately, converge on an increased risk of SCZ. Furthermore, early adaptations may remain latent until critical periods of normal maturation and neuronal pruning call into operation the damaged structures. Contrary to SCZ, the role of early environmental risk factors in BD is still less explored and will require further systematic longitudinal follow-up studies [38,39,40,41].

In any case, several findings indicate altered neurodevelopmental trajectories in BD: 

(i) Clinical risk factors such as a delayed development, neurocognitive and neurobehavioral impairments have been associated with poor prognosis in BD, especially in patients with early onset of disease [42]. Moreover, premorbid neurobehavioral impairments prevail in at risk individuals from offspring of first-degree relatives diagnosed with BD, who are more likely to manifest mood disorders [43]. Although premorbid alterations in attentional processes, cognition, and memory are common among both risk groups, further studies are needed to assess their role in the timing, progression, and course of BD.

(ii) Neuroimaging studies [44] support bilateral dysfunction in prefrontal-cortical hippocampal-amygdala emotion-processing and emotion-regulation circuits together with an overactive left-sided ventral striatal-ventrolateral and orbitofrontal cortical reward-processing circuitry. These dysfunctions associate with decreases in gray matter volume in the prefrontal and temporal cortices, the amygdala, and the hippocampus, and white matter tracts connecting prefrontal and subcortical regions. Considering that most of these investigations were conducted in adult case/control groups, future neuroimaging research in BD needs to be placed in the context of normative developmental and atrophic changes in neural structures and pathways thought to be involved in BD and focus on neurodevelopmental trajectories in youths with BD or at risk for BD [45].

(iii) Postmortem analysis of BD specimens has focused especially on the late maturing prefrontal cortex. Reduction in specific cortical layers comprise a reduced density of pyramidal cells in layers III and V [46], and of nonpyramidal neurons in layer II [47]. In the dorsolateral prefrontal cortex, these cellular alterations were less developed in BD than SCZ, while they appeared to be more prominent in other frontal and limbic structures of BD when compared to SCZ [46]. These cellular abnormalities could result in part from aberrant neocortical cell migrations as indicated by an increased presence of neuronal dysplasia and heterotopia in BD brain [48]. Different reports have suggested altered expression of factors involved in cortical cell migration such as Reelin [49], and DISC1 [50] in postmortem BD brains. Lastly, Golgi analysis of dendritic spines in neurons from the dorsolateral prefrontal cortex suggested reductions in spine densities, numbers of spines per dendrite, and mean dendrite length.

Overall, different lines of evidence point to altered neurodevelopmental trajectories in BD, which may be masked, at least in part, until adolescence when full-blown psychopathology manifests. Future longitudinal studies in individuals at risk are necessary to substantiate a role of early neurodevelopmental anomalies in BD.

## 5. The Promise of iPSCs

A decade ago, Takahashi et al. [51] made the transformative discovery that primary cells from any donor could be reprogrammed to a pluripotent state similar to that of cultured embryonic stem cells [52]. A bulk of studies has contributed to define the identity of iPSCs [53,54,55], to optimize their generation [56,57], and to explore their applications [58,59].

Ideally, each iPSC line contains the donor’s complete and unmodified genome and provides a unique tool for studying the relationship between the donor’s genotype and phenotype in vitro: (1) iPSCs can be differentiated into selected disease-relevant neuronal cell types including neurons and astroglia [60] and any of these cells are the result from expression of the donor’s genome; (2) iPSCs allow capturing critical neurodevelopmental transition phases that may be particularly sensitive to genetic perturbations; (3) iPSCs recapitulate many fundamental aspects of human neurodevelopment and associated disorders and provide adult-like cells of varying maturity [61]; (4) iPSCs provide limitless supply of anyone’s pluripotent cells in a dish and open up unprecedented opportunities to conduct functional studies in human brain related cell types, complementing previous approaches based on postmortem material and animal models; and (5) iPSCs can be derived from biobank samples of deceased persons for the investigation of multigenerational pedigrees.

Today, iPSCs are generated routinely from skin biopsies or PBMCs (peripheral blood mononuclear cells) [56,57]. Furthermore, refined in vitro trans-differentiation protocols allow the analysis of a large variety of different neuronal cells types [62]. This approach generates limited amounts of postmitotic neuronal cells and is cost-prohibitive for large scale integrated endpoint assays (e.g., transcriptomics, epigenomics, metabolomics, and proteomics) in a sufficiently powered first-line case/control design.

Collectively, iPSCs are a promising tool to explore molecular and cellular endophenotypes from polygenic BD in disease-relevant cell types.

## 6. BD in a Dish

During the last five years, an increasing number of studies have used iPSCs to gain insight into molecular and cellular alterations in BD. For clearness, we will group these studies by the common themes of their findings (Section 6.1, Section 6.2, Section 6.3 and Section 6.4) and summarize experimental approaches and key findings in a tabular format.

### 6.1. Neurodevelopmental Alterations in BD-derived iPSCs

The first report on BD did not appear before 2014: Chen et al. [63] established six quality controlled (Table 1) iPSCs lines (three BD vs. three controls), which were differentiated over eight weeks into forebrain-like neurons (Table 2).

Gene expression was assessed by microarrays and subsequent pathway analysis including KEGG (Kyoto Encyclopedia of Genes and Genomes) and gene ontology terms (Table 3). Transcriptomes from BD and control iPSCs differed little in the undifferentiated state and transcripts from various BD risk genes including *ANK3*, *CACNA1C*, *NCAN* (chondroite sulfate proteoglycan), *SYNE1,* and *ZNF804A* (zinc finger protein 804A) were well upregulated in neurons from both groups. In contrast, 140 transcripts were selectively increased 1.5-fold or higher in BD. These consisted of transcripts associated with cell matrix and/or cell–cell association and neuronal differentiation (axon outgrowth, synapse organization, secreted growth factors, neurotransmitters and their receptors, and membrane-associated channel genes, especially calcium channels). Another 95 transcripts were up-regulated by 1.5-fold or higher solely in control neurons and comprised genes contributing to cytoskeletal organization, cell surface/extracellular matrix factors, cell cycle, apoptosis, and DNA repair.

Beyond these general cellular processes, BD and control neurons differed specifically in the expression of genes guiding neural patterning: BD neurons showed higher expression of genes contributing to ventral differentiation including *FOXP2* (Forkhead Box P2) and *NKX2-1* (NK2 Homeobox 1). These encode transcription factors (TFs) with a defining role in the development of the medial ganglionic eminence. Conversely, control neurons showed higher expression of genes with a role in dorsal telencephalic neurons, including *PAX6* (Paired Box Gene 6), *EMX2* (Empty Spiracles, Drosophila, 2), *TBR2* (T-box, brain 2), *TCF3* (Trancription factor 3), and *ZNF 536* (zinc finger protein 536). Irrespective of these differences, iPSCs from both BD and controls remained responsive to patterning cues as addition of the ventralizing Hedgehog activator purmorphamine or the dorsalizing substance lithium stimulated expression of *NKX2-1* or *EMX2* (ventral or dorsal identity, respectively).

Neuronal-activity dependent TFs, especially those regulated by Ca^2+^ signaling, contribute to lineage differentiation [74,75]. Beyond this process, Ca^2+^ signaling impacts broadly synaptic plasticity. To simultaneously measure action potential and wave propagation, BD and control neurons were differentiated for another 4–8 weeks before loaded with the intracellular Ca^2+^ sensitive dye Fluo-4AM. By this time, cultures showed spontaneous neuronal firing. Of note, Ca^2+^ transient and wave amplitude were significantly reduced by lithium pretreatment in neurons from BD, but not from control iPSCs. Lithium dorsalizes early neural progenitors through activation of the wingless pathway [76] and may thus re-set NSC fate to dorsal cortical derivatives.

Taken together, neurons derived from BD iPSCs showed a bias for ventral cell fates though they remained responsive to dorsal cues and manifested hyperexcitability at mature stages. Both phenotypes were normalized by lithium pretreatment when compared to controls.

In a familial study on BD Madison et al. [65] investigated a subset of 4 individuals from a multigenerational kindred consisting of two affected brothers and their unaffected parents. Whole genome SNP profiling confirmed the expected parent-child and sibling relationships and further showed that the mother was heterozygous for the BD risk allele of *CACNA1C*, whereas the remaining family members were homozygous. Further, none of the family members showed gross chromosomal anomalies. iPSC quality control (Table 1) comprised SNP fingerprinting (to verify that iPSCs matched their donors), PluriTest (a bioinformatics approach that uses global gene expression profiles to bench mark unknown iPSCs with bona fide iPSCs) and teratoma formation in nude mice. Directed neuronal differentiation was used to produce NPCs (neural progenitor cells) that were immunopurified by a FACS (fluorescence activated cell sorting) protocol designed to remove non-neural and neural crest cells (i.e., peripheral nervous system progenitors) and to select for CXCR4 (CXC chemokine receptor-4) as a marker of central progenitors (Table 2). Interestingly, self-renewing NPCs could be easily obtained from the unaffected parents, whereas neural induction of iPSCs derived from the affected brothers consistently led to a higher number of neural crest progenitors. In support of this finding, BD NPCs showed a significant proliferation deficit when compared to the NPCs from the unaffected parents. This impairment was partly reversed by treatment with a selective GSK3β (glycogen synthase kinase 3) inhibitor; moreover, a general differentiation defect of BD iPSCs could be ruled out by NanoString (single-molecule imaging of color-added, molecularly barcoded probes) digital mRNA expression profiling on a panel of “scorecard” genes previously defined to assess quantitatively the differentiation capacity of embryonic stem cells/iPSCs [73].

In light of these results, Madison et al. [65] designed an additional set of gene probes consisting of pluripotency genes, patterning genes, and BD risk genes to gain insight into the neurodevelopmental deficit of BD-derived iPSCs. Expression profiles differed little in fibroblasts and iPSCs, but markedly in NPCs, in which 18 genes were differentially regulated when comparing unaffected parents with affected siblings. In BD-derived NPCs, three upregulated genes encoded homeodomain-containing TFs (*NKX2-2*; *NKX6-1*, NK6 homeobox 1; and *IRX3*, iroquois homeobox 3) with a known function in SHH (sonic hedgehog)-dependent ventralization of progenitor-derived neurons. Concurrently, several NPC-specific genes (e.g., *PAX6*, paired box gene 6; *DACH1,* dachshund homolog 1; *PLZF,* promyelocytic leukemia zinc finger protein; *ZBTB16,* zinc finger and BTB domain containing 16; and *DISC1*) were down-regulated in BD-derived NPCs.

Postmitotic neurons showed expression of multiple markers characteristic of upper and lower layer cortical projections neurons (e.g., CTIP2, COUP TF-interacting protein 2; CUX1, Cut-like homeobox 1; ETV1, Ets variant gene 1; SATB2, special AT-rich sequence-binding protein 2), among which *CTIP2* was downregulated in the affected children. This cortical layer marker is a component of NuRD (nucleosome remodeling and deacetylation complex) with a crucial role in neurogenesis [77] and in the differentiation of subcerebral projection neurons [78]. Similarly, RELN (reelin, an extracellular matrix glycoprotein) with a role in cortical neuron migration was downregulated in the affected children [49].

Besides neurodevelopmental genes, BD patient-derived neurons showed differential expression of several genes encoding calcium channels or other ion channel subunits: *CACNA1E* (encoding calcium channel, voltage-dependent, R-type, α1E subunit), *CACNA1G* (encoding calcium channel, voltage-dependent, T-type, α1G subunit), *CACNB1* (encoding calcium channel, voltage-dependent, β1 subunit), a regulator of α1 subunit membrane targeting and voltage dependent regulation, *CACNG8* (encoding calcium channel, voltage-dependent, γ subunit 8), the sodium channel genes *SCN2A* and *SCN3A* (encoding sodium channel, voltage-gated, α subunits) and a regulator of AMPA (α-amino-3-hydroxy-5-methy-4-isoxazolepropionic acid) receptor localization.

Lastly, a set of 53 genes was significantly upregulated (e.g., *SYN1*, *SCN2A*, *CNTN6* (contactin-6) and *NKX2.2*) or downregulated (*PAX6*, *DCX* (doublecortin), and *PLZF*) in both BD NPCs and postmitotic neurons. The latter three genes are typical markers of NPCs and thus strengthen the notion of an altered neurodevelopment of BD-derived iPSCs upon directed differentiation. 

Taken together, the findings from Chen [63] and Madison [65] on BD-derived iPSCs converge on common themes: (1) Impairments in cortical dorsal and central neuron/NPC formation; (2) Deregulation of genes with a critical role in cortical development, patterning, maturation, and Ca^2+^ signaling while risk genes from GWA studies show few, if any, alterations; and (3) Patterning and differentiation deficits can be partly rescued by lithium treatment pointing to aberrant Wnt signaling. Overall, these findings indicate altered neurodevelopmental trajectories in BD.

### 6.2. Neuronal Activity in BD-derived iPSCs

Immature neurons and networks express molecules and processes that are not operative in the adult and follow a crucial developmental sequence that is instrumental in the formation of functional entities [74]. Early alterations can delay or accelerate this developmental program (see above) and have thus sustained effects on brain architecture and electrical signatures that predate disorders to come well before clinical symptoms appear [79].

Mertens et al. [66] reprogrammed fibroblasts of six BD type 1 patients and four unaffected individuals using recombinant Sendai virus, a footprint free technology (Table 1). iPSC quality control consisted of ICC (immunocytochemistry), qRT-PCR (quantitative reverse transcribed polymerase chain reaction) analysis of pluripotency markers and of trilineage markers following random differentiation, and finally, Giemsa chromosome stains to assess karyotypes. Following differentiation into hippocampal dentate gyrus (DG) granule-like neurons, more than 80% corresponded to VGLUT1-positive glutamatergic neurons, while less than 7% were GABAergic. Glutamatergic and GABAergic neurons showed similar synapse densities independent of case/control status. By contrast, RNA-seq analysis detected 45 differentially expressed genes comprising multiple mitochondrial genes that showed a significant upregulation in diseased neurons. At the same time, BD mitochondria were smaller in size and showed an increased activity when compared to controls. Refined GO analysis of differentially expressed transcripts indicated an upregulation of the protein kinase A and C (PKA/PKC) signaling pathways and the action potential (AP) firing system in BD neurons when compared to controls. Well-fitting these findings, patch clamp analysis showed that BD neurons exhibited greater activation of Na^+^ channels, lower AP threshold, greater values of evoked AP number and maximal AP amplitude, and higher AP frequencies.

Since the investigated patients consisted of three lithium-responsive (LR) and three lithium nonresponsive (NR) patients, Mertens et al. further asked whether the hyperactive phenotype in iPSCs derived hippocampal neurons could be normalized by chronic lithium treatment. This was indeed the case; lithium significantly reduced Na^+^/K^+^ currents and the total number of evoked and spontaneous APs in neurons from LR patients. By contrast, lithium evoked no changes in neurons from NR patients that responded nevertheless efficiently to the Na^+^ channel antagonist lamotrigin. Hyperexcitability of BD neurons translated into increased neural network activity, which was partly normalized by lithium treatment of LR, but not of NR, BD patient-derived neurons. Lastly, RNA-seq analysis showed that the number of genes with significant expression changes in response to lithium treatment was an order of magnitude greater in the responding than in the nonresponding neurons (560 genes versus 40 genes). Among these genes were several that have been previously associated with BD (*PDE11A*, phosphodiesterase 11a; *PRKCH*, protein kinase C; *PTPRB*, protein tyrosine phosphatase B; *SCN11A*, sodium channel voltage gated type 11A; *NKAIN1*, Na^+^/K^+^ transporter ATPase-interacting 2; *KCNA1*, voltage gated potassium channel, shaker-related subfamily; and *KCNJ12*, inwardly rectifying potassium channel, subfamily J, member12) as well as 84 genes with a function in the PKA/PKC pathways, the AP firing system, and mitochondrial functions.

Collectively, these results suggest that iPSC-derived neurons from BD patients show an increased neuronal activity that was selectively reduced by lithium in neurons from those patients who were also responsive to clinical lithium administration. At the molecular level, neuronal hyperexcitability correlated with changes in the PKA/PKC/AP pathways and deregulation of mitochondrial genes in BD neurons.

These findings have been strengthened by a recent study by Stern et al. [69]: Epstein–Barr Virus-immortalized lymphocytes from LR and NR BD patients and healthy subjects were reprogrammed into iPSCs, quality controlled (Table 1), and differentiated into hippocampal dendate-gyrus like neurons (Table 2). About half of the cells expressed the Prox1 gene (Prospero-related homeobox 1), a marker for dendate granule cells, with no significant differences between patient and control samples. At 3.5 weeks of differentiation, patch-clamped Prox1-positive neurons from BD patients were hyperexcitable when compared to controls with the highest spontaneous firing group in the LR group. Moreover, in the voltage clamp mode, LR and control neurons showed similar sodium currents when compared to a decline by 45% in NR neurons. Consistent with their hyperexcitable phenotype, LR neuron spikes exhibited increased amplitude about 20% when compared with control neurons, while NR neuron spikes had 15% lower amplitude than control neurons. Lastly, these distinct features of BD neurons persisted from early to late time points of post differentiation, almost as if they were maintaining some distinct developmental program.

Going beyond basic cell culture studies, Stern et al. asked if a patient’s response to lithium could be predicted from the electrophysiological measurements of the corresponding iPSC-derived neurons. Therefore, a naïve Bayes classifier was fist trained with the electrophysiological features of neuronal populations from patients whose responses to lithium were known. Following this training period, the unknown lithium response of a new patient was accurately predicted (>92% success rate) by the classifier-based analysis of the respective iPSC-derived neurons.

Neuronal excitability reflects the aggregate state of many single variables: Hyperexcitable neurons within each group showed larger sodium and potassium currents that each opened at a less depolarized potential, larger amplitude narrower spike with a less depolarized threshold for evoking an action potential, and a very large increase in the fast afterhyperpolarization amplitude (AHP). In control neurons changes between hyper- and hyporesponsive neurons were most pronounced in the AHP. By contrast, changes in sodium currents prevailed in the NR group, whereas changes in the LR group were most prominent for sodium currents and AHP. These findings suggest that complex cellular phenotypes like neuronal hyperexcitability can be dissected into distinct subcomponents that differ between LR and NR BD patients and controls.

Consistent with previous findings [66], lithium treatment inhibited neuronal excitability in neurons from LR patients but not in neurons from NR patients. Hereby, the final effect of lithium on various electrophysiological parameters was quite similar in both LR and NR neurons: A decrease in the AHP and a spike broadening, making the cells less excitable, and an opening of sodium channels at lower potential, making the cells more excitable. This dual mode of lithium’s action could contribute to its effects of improving both depression and mania. Moreover, at the cellular population level, lithium shifted more excitable cells to the hyperexcitable state in the LR group, whereas in the NR group the shift seemed to operate in both directions leading to a similar number of cells that are hyperexcitable after lithium treatment. Thus, the effects of lithium on different cell types appeared to depend critically on their initial properties.

Overall, these studies suggest cell-autonomous, possibly developmentally driven, hyperexcitability in BD iPSC-derived neurons and neuronal networks. Hyperexcitability of LR and NR neurons involves distinct perturbations in their Na^+^/K^+^ channel activities and action potential parameters, with distinct electrophysiological changes after lithium treatment.

### 6.3. Lithium-Response Pathways in BD-derived iPSCs

Lithium has a unique role in the acute and maintenance treatment of BD and selectively ameliorates psychotic symptoms in mania, but not in SCZ. Lithium responsiveness is often regarded pathognomic of BD and has prompted a number of studies to interrogate lithium responsiveness in neurons derived from BD iPSCs (see also Section 6.2) in order to identify associated cellular phenotypes.

Recent advancements in reprogramming include the generation of neuronal cells either through an iPSC intermediate or via direct transdifferentiation of somatic cells, especially fibroblasts, to neurons [62]. Wang et al. [64] generated neuronal-like cells (iNLCs) from primary fibroblasts of LR or NR patients via transdifferentiation (Table 1). Experiments were carried out about two weeks after transduction when cells could be still passaged without severe damage. At this time, the neuronal phenotype as defined by axon and dendrite formation was still less developed and iNLCs expressed markers of NSCs, neurons, but also of fibroblasts, indicative of mixed cellular phenotypes and high cellular heterogeneity (Table 1).

iNLCs were passaged on the coated surface of a BIND biosensor that allows to quantitatively measure changes in cellular attachment as a shift in the resonated light. At the same time, cell morphology, including parameters like size, shape, and fraction of the sensor covered can be assessed. Interestingly, cells generated from LR donors adhered more strongly to the matrix than from NR donors, while control cells mapped in-between these two groups. Differences in cell adhesion were specific to the iNLCs and were undetectable in the matched fibroblasts supporting the utility of transdifferentiated neuron-like cells for identifying potential cellular correlates of lithium responsiveness. Moreover, none of the bipolar groups or of the control groups exhibited a significant difference in adherence following acute treatment with lithium. In light of the studies from above, the effect of chronic lithium treatment on integrin-mediated adhesion would be of considerable interest yet. Irrespective of this limitation, GWA studies on BD and SCZ have implicated genes with a role in cell adhesion [80,81,82] among which *ZNF804A* may operate in the functional coupling of the dorso-lateral prefrontal cortex across hemispheres and with the hippocampus [80].

**Figure 2:**
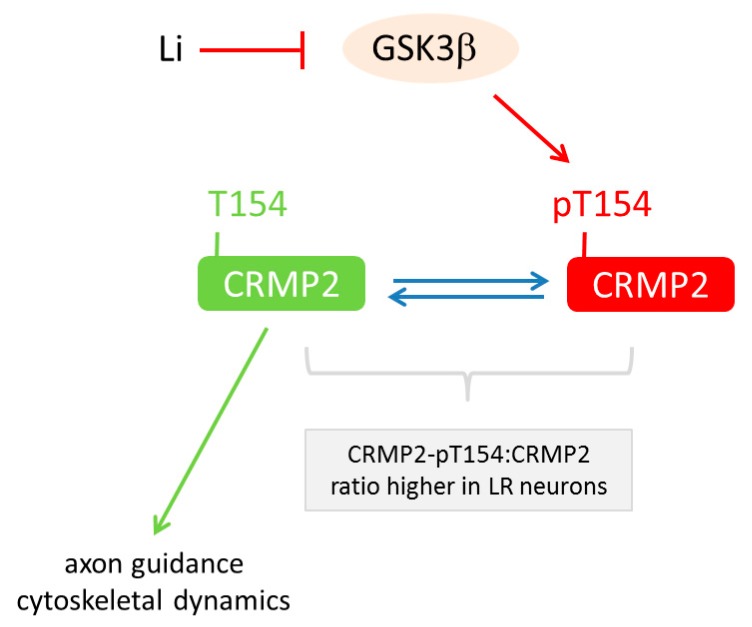
Model of the lithium-response pathway in bipolar disease. CRMP2 (collapsin response mediator protein-2) is a major target of GSK3β (glycogen synthase kinase). This enzyme catalyzes phosphorylation of the threonine (T) residue 154 in CRMP2. This step associates with CRMP2 inactivation in human neuronal progenitor cells and mature neurons. Conversely, inhibition of GSK3β by lithium prevents CRMP2 inactivation and supports its role in axon guidance and cytoskeletal dynamics. Interestingly, the ratio of CRMP2-pT154:CRMP2 is higher in neurons from lithium responsive (LR) patients and is normalized under lithium (Li) treatment.

Moving from cellular phenotypes to pathways, another study [70] focused on proteomic changes in anterior dorsal forebrain neurons generated from iPSCs derived from LR and NR donors, controls, and three patients with unrelated neuropsychiatric diseases (i.e., unipolar depression and Parkinson’s disease) (Table 2). Primary cells (fibroblast or lymphoblast) were reprogrammed through different techniques; quality assessed and differentiated into mixed GABA-ergic and glutamatergic interneurons (Table 2). Irrespective of diagnosis, neuronal differentiation was similar effective among all groups. Two-dimensional differential gel electrophoresis of lithium-treated and untreated BD-derived iPSC neurons detected 26 differentially represented proteins that corresponded to 15 distinct proteins as determined by mass spectroscopy. Among the respective genes, *CRMP2* (encoding collapsin response mediator protein-2) was found to be differentially expressed in postmortem dorso-lateral prefrontal cortices from BD patients when compared to controls. CRMP2 is a phosphoprotein with a well-known role in axon guidance and cytoskeletal dynamics by regulating the response to semaphorin 3A, an extracellular signaling molecule that binds to neurophilin/plexin receptors. Additional bioinformatic analysis indicated that CRMP2 may be a major target of GSK3β in lithium-responsive neurons from BD patients (Table 3). In support of this hypothesis, phospho-mapping in BD neurons showed that phosphorylation of threonine residue 514 (CRMP2-pT514) is the primary target of GSK3β-dependent lithium-action in human NPCs and mature neurons (Figure 2). While lithium reduced CRMP2-pT514 (i.e., the inactive form of CRMP2) in all iPSC-derived neurons irrespective of case/control status, baseline levels of CRMP2-pT514 were selectively higher in neurons from LR patients. Lithium application normalized CRMP2-pT514 to control levels indicative of an altered set-point regulation of CRMP2 phosphorylation in neurons from LR BD patients (Figure 2).

In support of these findings, CRMP2-pT514:CRMP2 ratios were elevated in samples from unmedicated postmortem BD brains concurrent with a decline in dendritic spine densities. Consistent with these results from SCZ iPSCs and postmortem brain, experiments in naïve and CRMP2-deficient knock-out mice supported a role in dendritic spine density [70].

Overall, this study shows that the lithium-response pathway in BD operates, at least in part, through GSK3β-dependent CRMP2 phosphorylation to alter the neuronal cytoskeleton, especially dendrite and dendritic spine formation, and presumably neural network development and activity. Thereby, the disturbed balance of CRMP2 phosphorylation may causally contribute to BD or result from the impact of altered neuronal signaling in LR BD patients.

### 6.4. Select Changes in Gene Expression in BD-derived iPSCs

Studies on iPSC-derived neurons from BD patients have analyzed RNA expression profiles with respect to neurodevelopment, neuronal activity, or lithium responses [63,65,66,69]. Here, we will discuss additional expression studies with a major focus on single genes or on familial BD.

Bavamian et al. [67] hypothesized that microRNAs (miRNAs) are of potential importance for BD given their high expression in the brain, their contribution to neuronal development, differentiation, and neuroplasticity [83,84,85], and their altered postmortem expression profiles in different psychiatric diseases including SCZ, autism, and anxiety [86]. miRNAs represent small non-coding RNAs of 18–23 nucleotides in size that regulate post-transcriptionally gene expression with each miRNA potentially controlling up to hundreds of downstream targets [87].

**Figure 3:**
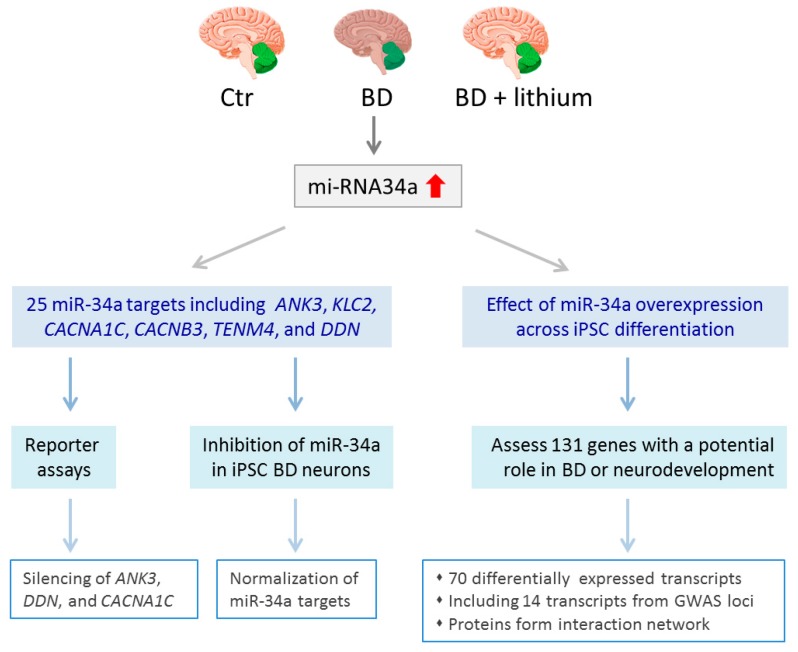
miRNA-34a in bipolar disease. miRNA-34a expression levels were selectively increased in the cerebellum from bipolar disease (BD) patients when compared to controls (Ctr) or patients that had received lithium treatment. Bioinformatic analysis predicted 25 miR-34a target genes including the GWAS loci *ANK3*, *KLC2*, *CACNA1C*, *CACNB3*, *TENM4*, and *DDN*. Transient reporter assays showed that miR-34a overexpression does silence *ANK3*, *CACNA1C*, and *DDN* expression. Conversely, inhibition of elevated miR-34a in neurons derived from BD iPSCs led to normalization of miR-34a target genes. In an orthogonal approach, Bavamian et al. [67] investigated the effects of miR-34a overexpression across iPSC differentiation on a panel of 131 genes with a potential role in BD or neurodevelopment. Among 70 differentially expressed transcripts, fourteen were encoded by GWAS loci. Furthermore, the corresponding proteins of these risk loci formed a highly connected protein interaction network.

Analysis of postmortem human brains from BD (*n* = 29) and controls (*n* = 34) showed that miRNA-34a was selectively increased in the cerebellum (Figure 3), but not in the prefrontal or anterior cingulate cortex, of diseased samples and was largely normalized in those patients that had received a mood stabilizer [67]. A pilot experiment with two iPSCs generated by Madison et al. (see above [65]) indicated an upregulation of miRNA-34a in the NPCs derived from the BD patient when compared with the unaffected father. To collaborate these findings, Bavamian et al. [67] transdifferentiated additionally fibroblasts from BD patients and controls into induced neurons and found that miRNA-34a was significantly upregulated in mature neurons from BD patients four weeks post-transduction (Table 1 and Table 2).

Bioinformatic analysis predicted 25 potential miR-34a targets among which 6 matched previous candidate genes identified through recent GWA studies: *ANK3*, *CACNA1C*, *CACNB3*, *TENM4*, *DDN* (dendrin), and *KLC2* (kinesin light chain 2). In agreement with the in silico analysis, miR-34a was capable of targeting and silencing *ANK3*, *DDN*, and *CACNB3* in transient reporter assays. Moreover, miR-34a targets were significantly down-regulated in iPSC-derived neurons of the BD patient. Conversely, inhibition of enhanced miR-34a expression in BD-derived neurons normalized expression of the downstream target genes. Going beyond direct miR-34a target genes, Bavamian et al. [67] also assessed via a customized NanoString assay the effects of miR-34a overexpression on 131 genes with a potential role in BD or neurodevelopment. A total of 70 transcripts showed a differential expression across iPSC differentiation among which 14 transcripts corresponded to genes associated with GWA study loci in BD including both *ANK3* and *CACNB3.* Furthermore, BD risk genes regulated by miR-34a overexpression formed a highly connected protein interaction network indicating that miR-34a coordinately controls pathways relevant to neurodevelopment and the expression of multiple candidate BD risk genes.

Taken together, this study suggests that the small non-coding RNA miR-34a is upregulated in BD and could affect the expression of multiple genes associated with BD, neurodevelopment, or neuroplasticity. In support of this hypothesis, increased miR-34a expression impaired the neuronal differentiation of BD iPSC-derived neurons.

In addition to altered neurodevelopmental pathways (Section 6.1), neuronal activity (Section 6.2), lithium response pathways (Section 6.3), and microRNAs (see above), a recent study [71] suggests an early onset of inflammatory processes in BD. Vizlin-Hodzic et al. generated six BD and four control iPSCs from adipocyte cells reprogrammed through episomal expression vectors (Table 1). Quality control comprised immunocytochemistry of pluripotency markers in undifferentiated iPSCs and of trilineage markers following undirected embryoid body formation. BD and control iPSCs differentiated with similar ease and efficiency into NSCs and then into functional cortical neurons of both excitatory and inhibitory type as demonstrated by whole patch clamp recordings. Few genes were differentially expressed in the iPSC stage, whereas 42 genes were significantly differentially expressed between BD and control derived NSCs and were enriched in various cytoskeleton-associated genes (*MAP7*, microtubulin-7; *TUBB8P7*, tubulin beta 8 class VIII pseudogene, *ANK1*, and *COL5A3*, collagen type V alpha-3). Of further note, *NLRP2* (NLR family, pyrin domain containing 2), encoding a protein with immune-regulatory functions, showed the greatest expression differences in both undifferentiated and neural cells, but not in the corresponding adipocyte cell lines, pointing to a potential role in early neurodevelopment.

NLRP2 (alias NALP2) contains an N-terminal pyrin domain and mediates activation of caspase-1 by Toll-like receptors, and possibly, also of protein complexes that activate proinflammatory caspases [88]. Moreover, both type I and type II interferons, as well as lipopolysaccharides (the Toll-like receptor 4 ligand) increase expression and secretion of NLRP2 and inhibit NFKB (nuclear factor kappa B) activation by a variety of stimuli [89]. In light of these biological function the identification of elevated *NLRP2* expression in BD iPSCs and NSCs is intriguing given perturbed immuno-inflammatory pathways in SCZ [90,91] and the shared genetics of BD and SCZ.

BD can be transmitted in a polygenic manner in extended pedigrees (Section 3) and iPSCs from affected families with a high penetrance of BD may provide a convenient tool to gain insight into disease associated cellular mechanisms. In an orthogonal approach to the genetic analysis of an Old Order Amish pedigree [18], Kim et al. [68] reprogrammed fibroblasts from type I BD patients and their unaffected siblings by transduction with Sendai virus (Table 1). Karyotypically normal iPSCs were differentiated into EBs and assessed by RT-PCR for the expression of genes specific for the three germ layers (Table 2). Following this, PAX6 positive NPCs were enriched from rosettes and differentiated into early (two-weeks) and late (four-weeks) cortical neurons. The latter showed a characteristic morphology consisting of axons, dendrites, and neurites, and stained positive for the pan-neuronal markers microtubule-associated protein 2 and β-tubulin III, the deep layer marker CTIP2, and the synaptic markers synapsin 1 and synaptophysin. Total RNA from NPCs, early and late neurons was investigated by microarray and principal component analysis and evidenced no changes in global gene expression across these stages compatible with a similar pattern and time course of differentiation between patient and control iPSCs. Moreover, there were no significant differentially expressed genes between BD and controls at the stage of NPCs or early neuronal differentiation. By contrast, late neurons showed upregulation of 292 genes in BD, while 32 genes were downregulated in sibling controls. Pathway analyses identified receptor-mediated signaling (e.g., Wingless, integrin, insulin, PI3K/AKT, IL-4, and hypoxia signaling, many of these involving GSK3β, a therapeutic target of lithium), RNA metabolism, and protein trafficking (e.g., post Golgi vesicle mediated transport and Golgi to plasma membrane transport) as major processes altered in BD-derived late neurons. Exemplary qRT-PCR confirmed the downregulation of the voltage gated type IV sodium channel beta subunit (SCN4B), a regulator of neuronal activity and neurodevelopment [92], and the upregulation of glutamate decarboxylase 1 (GAD1), a key enzyme for GABA synthesis, in late BD neurons (Table 3).

Taken together, this study suggests normal proliferation, but impaired late neuronal differentiation of iPSCs derived from familial BD patients. Additionally, upregulation of GAD1 in combination with downregulation of SCN4B points to a possible imbalance of excitatory and inhibitory neurotransmission in late neurons.

## 7. The Road Ahead: Promises, Caveats, and Challenges

iPSC-based disease modeling has led to the identification of a variety of potential neurodevelopmental and electrophysiological anomalies in BD-derived cells: Shifts in cell lineage decisions, deficits in cellular proliferation and adhesion, deregulation of mitochondrial function, inflammatory pathways, or miRNAs, and above all, alterations in Ca^2+^ and receptor-mediated signaling associated with increased neuronal excitability. Interestingly, some of these processes can be normalized by lithium treatment, a first line therapy in BD, and may recapitulate processes from the diseased brain. While altered differentiation potential and Ca^2+^ signaling are likely to influence each other [74,79], it remains to be clarified whether they entail shared or different genetic liabilities.

Notwithstanding this notable progress, we will consider next a number of caveats that may confound current findings and call for further improvements on the generation and design of patient-specific iPSC studies.

### 7.1. Karyotype Analysis

Several limitations are inherent to the iPSC technology and necessitate refined quality controls. Originally, reprogramming factors were introduced into fibroblast by means of retroviral virus vectors [51]. By integrating into the host genome, these vectors potentially cause genetic lesions confounding the analysis of molecular and cellular phenotypes in case/control studies. Therefore, non-integrating reprogramming methods comprising Sendai virus, episomal and mRNA transfections (Table 1) are the method of choice [93]. Each of these footprint-free technologies has different advantages and shortcomings with respect to reprogramming efficiency, reliability, workload, safety precautions, and costs. While traditional low-resolution karyotypic analyses (G-banding) can only identify large aneuploidies over 5 MB in size, Schlaeger et al. [93] used in a comparative reprogramming study an array system with an average resolution of 43 KB throughout the genome and 24 KB in RefSeq genes. This approach evidenced the highest aneuploidy for retroviral and episomal derived iPSCs (13.5% and 11.5% respectively), lower aneuploidy rates for lentiviral and Sendai virus-derived iPSCs (4.5% and 4.6% respectively), and the lowest aneuploidy rate for RNA-derived iPSCs (2.30%). These findings indicated that iPSCs may carry more mutations than other cultured somatic cells because of the reprogramming process and/or extended culture conditions. To address this topic, Kwon and coworkers [94] compared the mutational load of clonal fibroblast lines and iPSC lines generated from the same fibroblast. By means of whole exome sequencing they could show that iPSCs and clonal fibroblasts contain comparable number of new mutations, as compared with their parental (non-clonal) fibroblasts. Importantly, deep, targeted resequencing demonstrated that more than 90% of these mutations occur randomly and correspond to preexisting sequence variants in small subsets of the parental fibroblast population. In line with this finding we note that blood samples comprise multiple cell types including T or B lymphocytes that harbor pre-existing DNA rearrangements at the V(D)J locus and thus differ from the donor’s authentic genotype; a distinction that may influence the interpretation of immune-related processes in appropriate iPSC models. Alternatively, enrichment and reprogramming of hematopoietic progenitor cells provides a convenient source for patient-specific iPSC generation [95].

Irrespective of these caveats, there is now compelling evidence that common genetic variation underlies the molecular heterogeneity in iPSCs [96,97,98,99,100,101]. Genetic differences between individuals contribute significantly to transcriptional variation between iPSCs with donor effect accounting for a median of ~6% [97] and ~49% [101] of expression variation between iPSCs. Lesser donor specific effects of ~2% were reported from differentiated neuronal cells [102] possibly due to considerable variation in the differentiation capacity (see below). Furthermore, Kilpinen et al. [97] reported based on genome-wide profiling (RNA-sequencing, DNA methylation arrays, quantitative proteomics, and imaging of cell morphology) of a large number (*n* = 711) of systematically generated and genotyped iPSCs that 5–46% of the variation in different iPSCs phenotypes, including differentiation capacity and cellular morphology, relate to donor-specific effects. High resolution karyotype mapping (detection limit > 200 KB) of donor-matched samples also confirmed the existence of copy number alterations that need to be considered in case/control studies lending further weight to the results from Kwon et al. [94].

Taken together, donor-matched high-resolution karyotype maps are looked-for in reprogramming of BD specimens in order to detect and assess karyotypic anomalies more precisely. Since donor cell populations are usually non-clonal, multiple iPSCs should be generated, matched with the parental karyotype, and separately analyzed in functional studies. Moreover, due to the existing selection pressure under in vitro culture, iPSC karyotypes need to be routinely controlled for the elimination of growth promoting karyotypic anomalies that arise during long-term passage [94].

### 7.2. Differentiation Capacity

A major fraction of the iPSCs from the Kilpinen study [97] conformed to the criteria of pluripotency as deduced by PluriTest (84% and 97% score of over 20 and 10, respectively). This bioinformatic tool measures the transcriptional signature of pluripotency to classify samples with great sensitivity and specificity [72] and has been also used in the quality control of BD-derived iPSCs (Table 1). Virtually all iPSCs (over 99%) from the same study were successfully differentiated in the three germ layers albeit with some variability (70%, 84% and 77% of the cells expressing all markers of endoderm, mesoderm, and neuroectoderm, respectively). In an improvement to this two-step protocol, the ScoreCard approach (Table 1) measures in a single experiment the transcriptional signature of pluripotency and expression signatures that indicate functional pluripotency, defined by differentiation into each of the three germ layers [103]. Yet, none of these methods can predict the capacity for further differentiation towards distinct neuronal tissues and cell types that are sought in the analysis of BD-derived iPSCs. Differences in the neural differentiation capacity of human ESCs and iPSCs are well-known [73,104,105,106] and result from genetic variation that imparts a donor-specific expression and methylation profile in reprogrammed cells [107]. This is well in line with a recent study [101] on gene expression variability in 317 iPSCs from healthy donors, which suggested that ~49% of the genome-wide expression variability can be assigned to variation across individuals and partly derives from expression quantitative trait loci. Additionally, network, pathway, and key driver analysis revealed that Polycomb targets influence non-genetic variability seen within and across individuals and highlights the role of this dynamic chromatin regulator [108] in reprogramming-based variability [101]. Hence, donor-specific iPSC variability may bias differentiation propensity independent of disease status and poses a major challenge to case/control studies with a focus on early neural development (Table 3). Moreover, neural differentiation protocols depend on cumbersome and variable procedures as well as potentially instable pharmacological agents and bioactive proteins that introduce additional variations [109]. In agreement with these concerns, a recent large scale neuronal differentiation study of iPSCs (123 differentiations into sensory neurons) by Schwartzentruber et al. [110] showed that sample-to-sample variability in gene expression in iPSC-derived cells surpassed the one of their physiological counterparts (i.e., dorsal root ganglia) and indicated that genes thought to be relevant to the function of these cells are among the most variable. In fact, similar levels of variation were reported for neuron differentiation batch (24.7%) and donor and reprogramming effects in aggregate (23.3%). Because sample-to-sample variability was at least in part to the occurrence of varying mixture of cell types across differentiations, sorting by automated systems appears necessary to improve resolution of cellular phenotypes. Similar conclusions have been recently reached in an iPSC study on early on-set SCZ (14 patients and 12 controls) [102], which led the authors to conclude that decreasing intradonor expression variation by decreasing cellular heterogeneity in neuronal differentiation protocols is critical to distinguish between case/control samples.

Collectively, these findings pinpoint current caveats in the assessment of neuronal differentiation capacity. As a minimum requirement, multiple karyotypically intact iPSC lines derived from the same donor need to be investigated and refined differentiation protocols are looked for to reduce intra- and inter-donor cellular heterogeneity during directed differentiation in case/control studies and across different studies.

### 7.3. Study Size

Mendelian disorders harboring rare, highly penetrant coding variants with large phenotypic effects are ideal candidates for iPSC-based disease models and require small cohort sizes. By contrast, studying common mostly non-coding variants of modest effect size from complex diseases (Section 3) will require minimizing biological and technical variance. While automated, high-throughput protocols for the generation and characterization of iPSCs and embryoid bodies have been developed [111], subtlety and workload of present neural differentiation protocols remain critical and makes them less suitable to automatization. Despite such limitations, Schwartzentruber et al. succeeded in their large scale iPSC study [110] in the detection of thousands of quantitative trait loci that influenced gene expression, chromatin accessibility, and RNA splicing during neuronal differentiation. Based on these results the authors hypothesize that iPSCs derived from 20–80 individuals are sufficient to enable the detection of the effects of common regulatory variants with moderately large effect sizes. Cohort sizes may be reduced without losses in power by future advancements on neuronal differentiation in combination with FACS-sorting or single-cell sequencing of mixed neuronal populations [102,110].

Taken together, these findings indicate that cohort sizes of current BD iPSCs studies should be enlarged, while the size of cohorts that is needed to resolve the smaller effects of common variants remains still uncertain. 

### 7.4. Conclusions

BD continues to present a global challenge to mental health and more people are affected every year. There is a tremendous need to understand the underlying pathophysiology of this severe condition and to develop new, tailored treatments [112]. iPSC-derived neurons firstly enable the study of molecular mechanisms of BD in relevant human cell types, including those that are inaccessible as primary tissue samples. While the suitability of iPSCs for the study of complex human conditions like BD has not been fully explored, preliminary evidence supports a role of early developmental alterations in BD that comprise neural patterning, Ca^2+^ and receptor-mediated signaling, and increased postmitotic neuronal excitability. Generation of iPSCs has made substantial progress over the last decade and is nowadays routine work and scalable. By contrast, variability in neuronal differentiation is still poorly understood and represents a critical bottleneck in the detection of smaller effects within the size of cohorts that can be realistically assembled. Hence, progress on technical and bioinformatic tools in the analysis of mixed neuronal populations will be necessary to fully exploit the potential of iPSC-based disease modeling for BD processes and eventually, the development of new treatments.

## Figures and Tables

**Table 1:** Methods for iPSC generation and quality control in BD studies.

Ref.	Source	Factors	Methods	N°	Auth	Karyo	Pluripotency
[63]	FB	OKSM	RV	≥5	nd	nd	ICC, EB and TriL
[64]	FB	miR/NAM	LV	na	na	na	ICC
[65]	FB	OKSM	RV	≥3	SNP	G-Band	ICC, PluriTest, EB and ScoC, Tera
[66]	FB	OKSM	SV	2	nd	G-Band	ICC, TriL
[67]	FB	miR/NAM	LV	na	na	na	ICC
[68]	FB	OKSM	SV	3	nd	G-Band	ICC, EB and TriL
[69]	LCL	OKSM, LIN28	Epi	≥3	STRP	nd	ICC
[70]	FB, LB	OKSM	Epi, LV, RV	1–3	SNP	nd	ICC, PluriTest, EB, Tera
[71]	AP	OKSM	Epi	1	nd	nd	ICC, EB and TriL

AP, adipocytes; Auth, authentication; EB, undirected embryoid body formation; Epi, episomal plasmid; FB, fibroblast; G-Band, chromosomal G-banding; ICC, immunocytochemistry; Karyo, karyotype; LB, lymphoblast from blood; LCL, lymphoblastoid cell line; LV, lentiviral transduction; miR/NAM, miR9/9*-124, NEUROD2, ASCL1, and MYT1L; OKSM, OCT4, KLF4, SOX2, MYC; N°, numbers of independent clones per donor; na, not applicable; nd, not determined; PluriTest, a bioinformatic approach to asses pluripotency [72]; Tera, teratoma formation; TriL, analysis of trilineage formation by ICC and/or qRT-PCR; Ref, reference; RV, retroviral transduction; ScoC, lineage score card [73]; SNP, whole genome single nucleotide profiling; SV, Sendai virus transduction; STRP, short tandem repeat profile.

**Table 2:** Study design, cellular model, and neuronal cell types.

Ref.	Study Design	BD vs. Controls	Model	Major Cell Type(s)
[63]	BD I (LR)—control	3 vs. 3	iPSC	Forebrain, mixed glutamatergic—GABAergic neurons
[64]	BD I—control	12 vs. 6	iNLC	NSC, NPC, neuronal-like cells
[65]	Familial BD	2 vs. 2	iPSC	FACS-sorted NPCs, neurons
[66]	BD I—control	6 vs. 4	iPSC	Hippocampal dentate gyrus-like granule neurons
[67]	BD I—control	1 vs. 1 5 vs. 3	iPSC iNLC	NPC, NPC-derived neurons transdifferentiated neurons
[68]	Familial BD	4 vs. 4	iPSC	PAX6-positive NPC, early and late cortical neurons
[69]	BD I (LR-NR)—control	3 vs. 3 vs. 4	iPSC	Hippocampal dentate gyrus-like granule neurons
[70]	BD I (LR-NR)—control—MDD—PD	7 vs. 3 vs. 6 vs. 2 vs. 1	iPSC	Forebrain, mixed glutamatergic—GABAergic neurons
[71]	BD I—control	6 vs. 4	iPSC	Cortical stem and progenitor cells

BD I, bipolar disease type I; FACS, fluorescence activated cell sorting; iNLC, induced neuron-like cells; iPSC, induced pluripotent stem cell; MDD, unipolar major depression; LR, lithium responder; NPC, neural progenitor cell; NSC, neural stem cell; NR, lithium non-responder; PD, Parkinson disease.

**Table 3:** Major analytical methods and findings in BD-derived iPSCs.

Ref.	Major Methods	Major Findings in BD-derived iPSCs
[63]	Microarray, Ca2^+^ transients	Ventralization, increased expression of membrane bound receptors and ion channels, Li reduces wave altered length amplitude and Ca^2+^ transients
[64]	Morphology, Res-Imag	Cellular adhesion associates with clinical response to Li
[65]	NanoString, RNA-seq, WCPC	Impaired early NPCs proliferation that is normalized by GSK3β inhibitor, altered WNT/GSK3β signaling and ion channel expression in NPCs
[66]	RNA-seq, WCPC	Altered neuronal excitability, altered mitochondrial function and size, Li reduces hyperexcitability in LR donors and partly normalizes mitochondrial function
[67]	qRT-PCR, NanoString, reporter assays	Upregulation of miR-34a in NPC and neurons, reducing miR-34a expression enhances dendritic elaboration and maturation of NPCs
[68]	Microarray	Deregulation of receptor-mediated signaling. RNA metabolism, and protein trafficking in late neurons, upregulation of GAD1
[69]	WCPC	Neurons differ according to LR and NR, larger fast after-hyperpolarization
[70]	Proteomics	Li-response pathway in BD acts through GSK3β-dependent CRMP2 phosphorylation to alter dendrite and dendritic spine formation, Ca^2+^ fluxes and neuronal activity
[71]	RNA-seq, WCPC	Upregulation of immune-regulatory NLRP2, GABA- and dopamine signaling

CRMP2, collapsin response mediator protein-2; GAD, glutamate decarboxylase; GSK3β, glycogen synthase kinase 3; Li, lithium; LR, lithium responder; miR-34a, microRNA-34a; NanoString, digital expression profiling; NPC, neuronal progenitor cell; NR, lithium non-responder; NLRP2, NLR family pyrin domain containing 2; qRT-PCR, quantitative reverse transcribed real-time polymerase reaction; Res-Imag, resonance imaging; RNA, ribonucleic acid; RNA-seq, RNA sequencing; WCPC, whole cell patch clamp; WNT, Wingless-type MMTV integration site family.
